# The Metastasis Suppressor, N-myc Downregulated Gene 1 (NDRG1), Is a Prognostic Biomarker for Human Colorectal Cancer

**DOI:** 10.1371/journal.pone.0068206

**Published:** 2013-07-09

**Authors:** Zhihai Mao, Jing Sun, Bo Feng, Junjun Ma, Lu Zang, Feng Dong, Daohai Zhang, Minhua Zheng

**Affiliations:** 1 Department of General Surgery, Ruijin Hospital, Shanghai Jiao Tong University School of Medicine, Shanghai, People’s Republic of China; 2 Department of Pathology, University of Sydney, Sydney, New South Wales, Australia; 3 Shanghai Minimally Invasive Surgery Center, Shanghai, People’s Republic of China; The Chinese University of Hong Kong, Hong Kong

## Abstract

Metastasis remains to be one of the most prevalent causes leading to poor long-term survival of colorectal cancer (CRC) patients. The clinical significances of tumor metastatic suppressor, N-myc downregulated gene 1 (NDRG1), have been inconsistently reported in a variety of cancerous diseases. In this study with 240 CRC clinical specimens, we showed that NDRG1 expression was significantly decreased in most of CRC tissues compared to the paired non-tumor counterparts. Statistical analysis revealed a significant inverse correlation of NDRG1 expression with tumor stage, differentiation status and metastasis. Compared with NDRG1-negative group, NDRG1-positve group had better disease-free/overall survival (*p* = 0.000) over 5 years’ follow-up. Furthermore, NDRG1 was considered to be an independent prognostic factor for overall survival (*p* = 0.001) and recurrence (*p* = 0.003). Our study concludes that NDRG1 is a novel favorable predictor for the prognosis in CRC patients.

## Introduction

Colorectal cancer (CRC) is one of the most common malignancies worldwide [1]. It has been shown that at the early stage, CRC can be cured by minimally invasive radical surgical resection [2]. However, a large population of CRC patient has been diagnosed at an advanced stage and conventional treatment options become unavailable [3]. Moreover, CRC represents an aggressively invasive and metastatic tumor type [4] and metastatic recurrence remains to be one of the most prevalent causes leading to poor long-term survival and high mortality rate [5]. Therefore, understanding the molecular pathogenesis of cancer recurrence and metastasis will help to improve the treatment regimes and disease prognosis for CRC patients.

Colorectal carcinogenesis is a complicated process involving numerous protooncogenes and tumor suppressor genes [6]–[9]. We and others have found that aberrant expression of a novel metastasis suppressor, N-myc downregulated gene 1 (NDRG1), was involved in the process of CRC development [9]–[12]. NDRG1 exerts its function through modulating the major signaling pathways in a large variety of tumor types including CRC [9], [10], [13]–[15]. Nevertheless, the association of NDRG1 with cancer progression has been reported with inconsistent observations. For instance, NDRG1 expression in breast, liver, lung and cervical cancers was positively correlated to the disease relapse and was a poor prognostic indicator for the patient survival [16]–[20]. On the contrary, NDRG1 expression in prostate, colon and esophageal tumors was a favorable factor for patient clinical outcome [21]–[23]. Apparently, the clinical role of NDRG1 in cancerous disease occurs in a context-dependent manner.

The clinical significances and prognostic values of NDRG1 in CRC have not been well evaluated. Traditionally, the tumor infiltration depth, the number of metastatic lymph nodes, tumor location, distant metastasis and completeness of excision, and the levels of postoperative serum carcinoembryonic antigen (CEA) and carbohydrate antigen 19-9 (CA19-9) have been used as indicators of prognosis, which are not fully reliable yet [24]. Therefore, developing new prognostic biomarkers will be of significance to guide the treatment of CRC. Recently, a number of putative biomarkers have been developed to predict the response to specific adjuvant treatment. These biomarkers include proliferating cell nuclear antigen (PCNA), CEA, CA19-9, p53, Kirstenrat sarcoma-2 virus oncogene (K-ras), Microsatellite instability (MSI), vascular endothelial growth factor (VEGF) and *etc*. [25]. From the clinical point of view, their prognostic and therapeutic significances have not been fully validated [25]. In this study, the expression of NDRG1 and its clinicopathological significances were investigated in 240 CRC specimens and their paired non-tumor counterparts. Our studies demonstrated that NDRG1 is a novel favorable biomarker for long-term survival in CRC patients.

## Materials and Methods

### Patients Selection

Patients who underwent laparoscopic surgery for colorectal cancer were consecutively enrolled from January 2006 until December 2007. The trial was approved from Research Ethics Committee of Ruijin Hospital, Shanghai Jiao Tong University School of Medicine, and the written informed consent obtained from all patients involved prior to this study. None of the patients enrolled had been accepted preoperative radiotherapy or chemotherapy. Preoperative cancer staging was performed by enhanced CT scan. All patients enrolled are eligible to be treated by laparoscopic approach after preoperative evaluation. All laparoscopic procedures were performed by the same surgical team. All surgical operations followed a standard D2 lymph node dissection protocol according to the Guidelines of Radical Laparoscopic Colorectal Cancer Surgery (2006) established by the Study Group of Laparoscopic and Endoscopic Surgery Affiliated to Chinese Medical Association. All CRC patients who were pathologically diagnosed as stage III–V accepted adjuvant chemotherapy postoperatively. Exclusion criteria were: *in situ* disease, emergency presentation, body mass index (BMI)>35 kg/m^2^, American Society of Anesthesiologists (ASA) classification IV-V, associated gastrointestinal disease that required extensive operative evaluation or intervention, pregnancy or malignant disease in the past 5 years (except superficial squamous or basal cell carcinoma of the skin or *in situ* cervical cancer). Postoperative clinical staging was based on the UICC cancer staging manual (7th edition, 2009), evaluating *via* preoperative enhanced CT scan, intraoperative discoveries together with postoperative pathological evaluations.

### Immunohistochemistry (IHC)

Tumor tissues and the paired non-tumor tissues at the resection margins were collected, fixed with formaldehyde and embedded with paraffin. IHC staining was performed as previously described [26]. After permeabilization and antigen-retrieval, the sections were incubated with anti-NDRG1 antibodies (HPA006881, Sigma-Aldrich, St Louis, MO, USA) at 4°C overnight, followed by incubation for 1 h/20°C with the horseradish peroxidase (HRP)-conjugated secondary antibody (Sigma-Aldrich). Negative controls were obtained by the addition of blocking peptide to the primary antibody. The sections were then washed with TBS and treated with the 2-Solution DAB Kit (Invitrogen, Camarillo, CA, USA) according to the manufacturer’s procedure. The tissues were counterstained with Mayer’s hematoxylin.

### Scoring of IHC Staining Results

All the IHC sections were examined and scored under a light microscope (Olympus, Tokyo, Japan) by a pathologist and the principal researchers. Cases with discrepant scores were rescored by the same or additional scorers to obtain a consensus score. NDRG1 scoring was done according to the widely-used German semi-quantitative scoring system, taking into account the staining intensity and the percentage of stained tumor cells [27], [28]. Staining levels were scored as 0 (no staining), 1 (weak staining), 2 (moderate staining) and 3 (strong staining), based on the staining intensity in the tumor cells. The percentage of stained tumor cells in each section was counted and the sections were scored accordingly (<10% = 0, 10–25% = 1, 26–50% = 2, 51–75% = 3, 76–100% = 4). The final immunostainning score of each tumor tissue section was determined by multiplying the intensity scores with the scores of positively stained tumor cells, with the minimum score of 0 and a maximum score of 12. Tumor sections with score 1–4 were considered as negative, whereas tumor sections with score 5–12 were considered as positive.

### Protein Extraction and Immunoblotting

Fresh cancerous tissues from 10 CRC patients (stage III-IV) and the paired non-tumor counterparts were harvested and the total proteins were extracted. Immunoblots were performed according to the established protocols [10]. The expressions of NDRG1 and GAPDH were assessed with primary antibodies against NDRG1 (HPA006881, Sigma-Aldrich) and GAPDH (sc-32233; Santa Cruz, CA, USA), followed by incubation with HRP-conjugated anti-rabbit (A9169) and anti-mouse (A4416) antibodies (Sigma-Aldrich). The chemiluminescent signals were visualized and captured with the BioImaging System (BIO-RAD, Hercules, CA, USA) and analyzed using the Image Lab™ Software Version 4.0.1 (BIO-RAD). The experiments were repeated at least three times independently. The relative NDRG1 expression levels in triplicate experiments were normalized to the level of GAPDH.

### Postoperative Follow-up Evaluation

All patients were followed-up after being discharged from the hospital until the date of 10th December, 2012. Recurrences were confirmed clinically or histologically if distant metastasis, locoregional relapse (tumor growth restricted to the anastomosis or the region of primary operation) and incisional metastasis were detected. The duration from the date of operation to the date indicating the last follow-up evaluation, treatment failure/recurrence or death was recorded.

### Statistical Analysis

Data were collected prospectively using a computerized database according to pre-study *Power* calculation. Quantitative data were accessed by using Student’s *t*-test. Count data were assessed by Mann-Whitney, Chi-Square or Fisher’s exact test when appropriate. Recurrence and overall survival were evaluated using the Kaplan-Meier method and compared by log-rank test. Analysis of predictive factors for survival was performed and the variables associated with recurrence and survival were then used for multivariate analysis using a stepwise Cox proportional-hazards regression model. Statistical significance was defined as *p*<0.05. All calculations were performed with the SPSS software package version 12.0 (SPSS Inc., Chicago, IL, USA).

## Results

### Demographic, Anatomopathological and Clinical Data

A total of 240 patients were enrolled in the study. The mean age of the onset of the disease was 68, with most patients ranged in age between 60 and 80 (61.67%) (Table 1). There were 132 (55%) male patients and 108 (45%) female patients involved in this study, with BMI 21.6±5.8 kg/m^2^ (16.3–31.7) (Table 1). Among these 240 patients, 136 patients were combined with perioperative comorbid disease (Table 1). In addition, the tumor location in the study population was also shown in Table 1.

**Table 1:** Demographic and clinical data.

		Patients Enrolled (N = 240)		
Parameters		n	%	Range
**Age (Years, mean ± SD)**		68±13		22–90
	**20–39**	8	3.3	
	**40–59**	50	20.83	
	**60–79**	148	61.67	
	**≥80**	34	14.17	
**BMI (kg/m^2^, mean ± SD)**		23.6±3.4		15.8–32.6
	**<18**	8	3.33	
	**18–24**	118	49.17	
	**≥24**	114	47.50	
**Gender**	**Male**	132	55.00	
	**Female**	108	45.00	
**ASA score**	**I**	18	7.50	
	**II**	126	52.50	
	**III**	96	40.00	
**Perioperative Comorbid Diseases**	**No**	104	43.33	
	**Yes**	136	56.67	
	**Cardiovascular**	36		
	**Respiratory**	20		
	**Hepatic Cirrhosis**	12		
	**Renal Failure**	6		
	**Cerebral Infarction**	22		
	**Diabetes Mellitus**	42		
	**Autoimmune**	6		
	**Others**	12		
**Tumor Location**	**Right-hemi Colon**	68	28.33	
	**Transverse Conlon**	4	1.67	
	**Left-hemi Colon**	16	6.67	
	**Sigmoid**	54	22.5	
	**Rectum**	98	40.83	
**Perioperative Mortality**		0	0	

Investigations towards postoperative specimens indicated that the proximal resection margin was 12.3±5.9 cm and the distal resection margin was 10.8±4.1 cm in colon cancer cases. In rectal cancer cases, the proximal resection margin was 9.1±2.7 cm and the distal resection margin was 5.2±1.9 cm (Table 2). Resection margins were measured in fresh specimens after surgery without fixation. The postoperative pathological confirmation indicated that there was no case with positive resection margin. Moreover, the lymph nodes were retrieved from each sample after fixation and the number of the lymph nodes retrieved was 14±6 (Table 2). These results supported the effect of the standardized operative procedures in this study.

**Table 2:** Anatomopathological data.

		Patients Enrolled (N = 240)		
Parameters		n	%	Range
**Tumor Size (cm, mean ± SD)**		4.6±1.7		1.5–9.0
	**<5**	134	55.83	
	**≥5**	106	44.17	
**Proximal Margin for colon cancer (cm, mean ± SD)**		12.3±5.9		6–21
**Distal Margin for colon cancer (cm, mean ± SD)**		10.8±4.1		6–18
**Proximal Margin for rectum cancer (cm, mean ± SD)**		9.1±2.7		6–15
**Distal Margin for rectum cancer (cm, mean ± SD)**		5.2±1.9		3–8
**Lymph Node Retrieved (mean ± SD)**		14±6		6–38
**Differentiation**	**Well**	26	10.83	
	**Moderate**	140	58.33	
	**Poor**	34	14.17	
	**Mucinous**	40	16.67	
**pT**	**pT1**	10	4.17	
	**pT2**	40	16.67	
	**pT3**	104	43.33	
	**pT4**	86	35.83	
**pN**	**pN0**	154	64.17	
	**pN1**	62	25.83	
	**pN2**	24	10.00	
**pM**	**pM0**	216	90.00	
	**pM1**	24	10.00	
**TNM Stage**	**I**	30	12.50	
	**II**	124	51.67	
	**III**	62	25.83	
	**IV**	24	10.00	

Postoperative pathological staging were performed by a group of experienced pathologists. The mean tumor size was 4.6±1.7 cm (1.5–9.0 cm) (Table 2). The tumors were defined as well-differentiated in 26 cases (10.83%), moderately-differentiated in 140 cases (58.33%), poorly-differentiated in 34 cases (14.17%) and mucinous carcinoma in 40 cases (16.67%) (Table 2). The evaluations of tumor infiltration (pT), lymph node metastasis (pN) and metastasis (pM1) were shown in Table 2. In total, the clinical TNM staging indicated that 30 cases (12.5%) were in stage I, 124 cases (51.67%) in stage II, 62 cases (25.83%) in stage III and 24 cases (10%) in stage IV (Table 2).

All patients were followed-up in line with the proposed postoperative surveillance protocol. The total follow-up duration was 3.8–83.9 months, with a median follow-up time of 67.4 months. Of all the enrolled patients, 72 cases (30%) were confirmed as postoperative recurrence (Table 3). Furthermore, the overall mortality rate was 28.33% (68 cases), in which 66 cases (27.5%) were tumor-related and two deaths were caused by acute cerebral hemorrhage and respiratory dysfunction, respectively (Table 3). In these 66 cases, 45 cases (68%) were NDRG1 negative and 21 cases (32%) were NDRG1 positive (p = 0.000). No significant relations between NDRG1 and the clinicopathological criteria were observed in this “death group” (Table 4).

**Table 3:** Postoperative recurrence and mortality data.

		Patients Enrolled (N = 240)	
		n	%
**Overall Recurrence**		72	30.00
**Type of Recurrence**	**Locoregional Relapse**	28	11.67
	**Metastasis**	42	17.50
	**Incision-related**	2	0.83
**Overall Mortality**		68	28.33
**Cause of Death**	**Tumor-related**	66	27.5
	**Others**	2	0.83

**Table 4:** Association of NDRG1 with clinicopathological criteria in tumor-related “death group”.

		Cancer-related Deaths (N = 66)		
Parameters		NDRG1 (+)(n = 21)	NDRG1 (−)(n = 45)	*p*
**pT**	**pT1**	0	0	0.095
	**pT2**	2	6	
	**pT3**	6	23	
	**pT4**	13	16	
**pN**	**pN0**	3	7	0.408
	**pN1**	10	13	
	**pN2**	8	25	
**pM**	**pM0**	11	31	0.059
	**pM1**	10	14	
**TNM stage**	**I**	2	6	0.389
	**II**	1	1	
	**III**	8	24	
	**IV**	10	14	

### NDRG1 Expression was Reduced in CRC Tissues Comparing to the Paired Non-tumor Tissues

**Figure 1:**
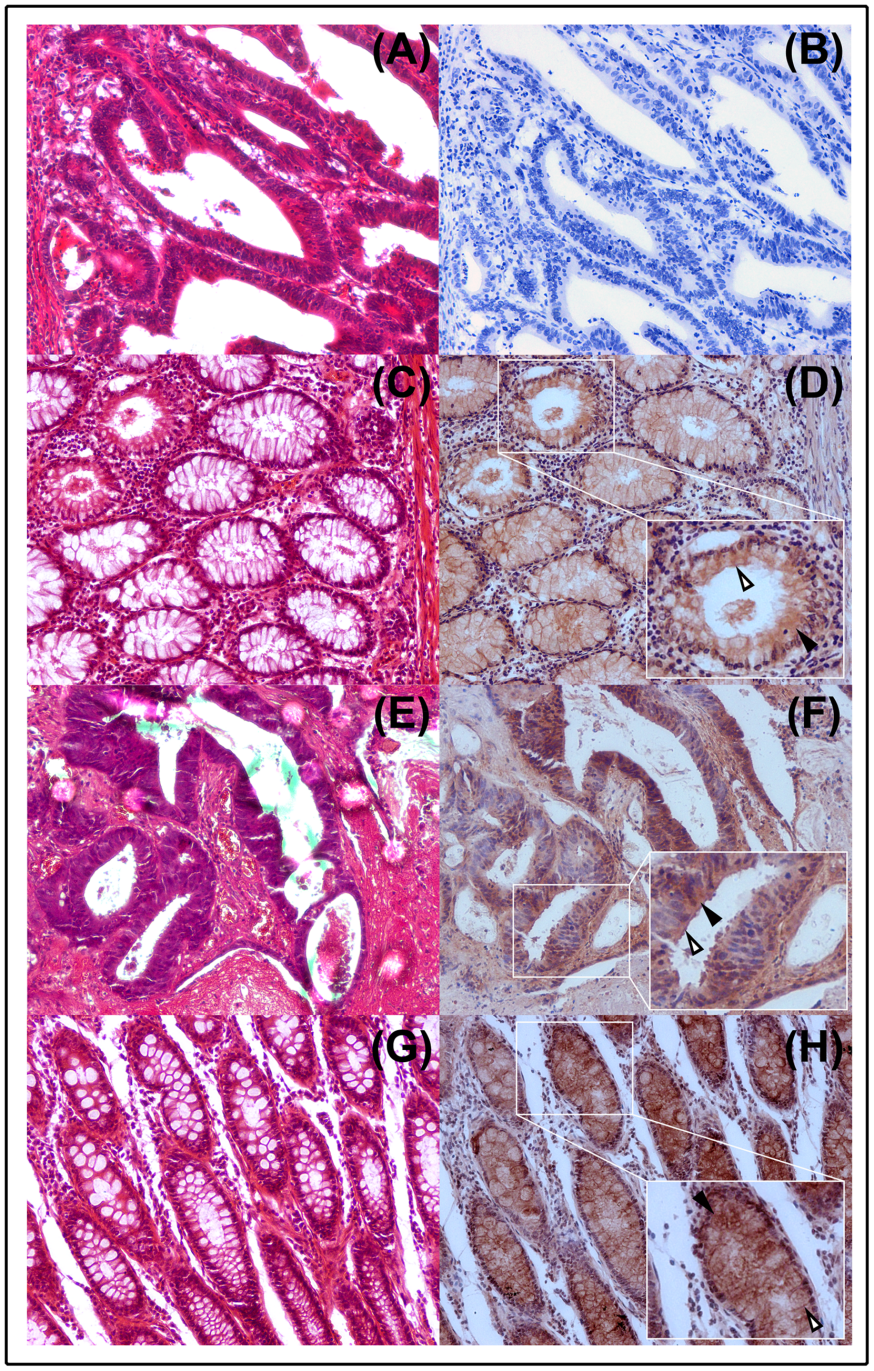
HE and immunohistochemical staining of NDRG1 in colorectal cancer and relative normal tissues. (A–D) Representative samples with HE staining (A) and NDRG1 negative expression (B) in cancer tissue, as well as HE staining (C) and NDRG1 positive expression (D) in the paired non-tumor tissue. (E–H) Representative samples with HE staining (E) and NDRG1 positive expression (F) in cancer tissue, as well as HE staining (G) and NDRG1 positive expression (H) in the paired non-tumor tissue. The black arrows indicate NDRG1 expression in cytoplasma, while the hollow arrows indicate NDRG1 expression in cell membrane. Magnification, 200×.

NDRG1 expression in CRC and non-tumor tissues was examined by IHC and immunoblot. IHC staining indicated that NDRG1 was predominantly presented in the cytoplasm in CRC cells and normal cells in the paired non-tumor colorectal tissues. NDRG1 expression was also observed at the cell membrane (Figure 1). In CRC specimens, NDRG1 expression was negative in 104 cases (43.33%), and positive in 136 cases (56.67%). However, in the non-tumor tissues, NDRG1 showed a high expression in 196 cases (81.67%) and negative expression in 44 cases (18.33%). This indicated that reduction or loss of NDRG1 may facilitate tumorigenesis in colon.

**Figure 2:**
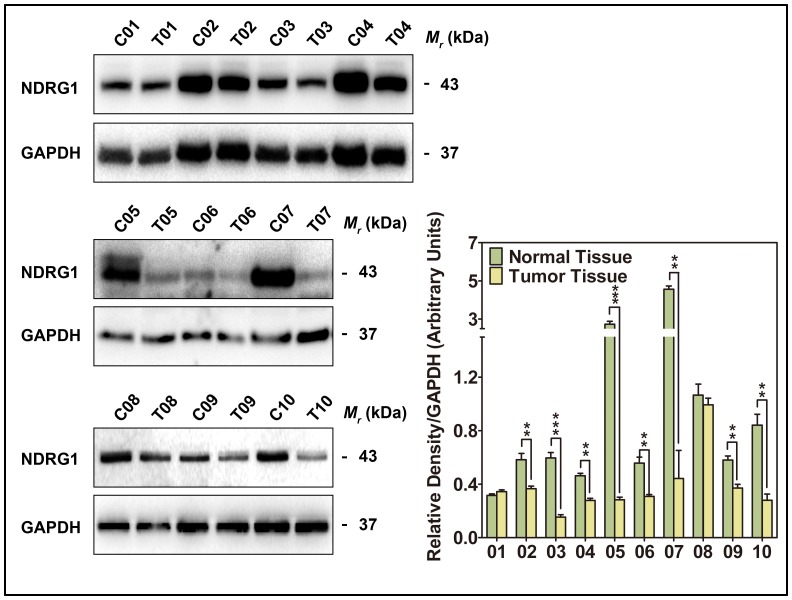
Immunoblot of NDRG1 expression in 10 pairs of advanced CRC tissues. C: Non-tumor tissue; T: Tumor tissue. NDRG1 levels were expressed relative to the loading control, GAPDH. Data are typical of 3–5 experiments and the histogram values are mean ± SD. ** *p*<0.01, ***p<0.001, relative to respective control group.

NDRG1 expression was also analyzed by immunoblot in 10 CRC tumors at stage III-IV and 10 paired non-tumor tissues (Figure 2). Comparing to the level of NDRG1 in the non-tumor tissues, 8 CRC tissues showed a significant reduction of NDRG1 (*p*<0.001–0.01). Only 2 CRC tissues expressed a similar level of NDRG1 to that observed in the paired non-tumor tissues. Overall, these data further supported a decrease of NDRG1 expression in advanced CRC tissues.

### NDRG1 Expression in CRC Correlates with Patient Outcomes

Since the expression of NDRG1 in CRC tissues was significantly decreased comparing to that in non-tumor tissues, the correlations between NDRG1 expression in CRC and the clinical characteristics were analyzed and summarized in Table 5. NDRG1 expression was detected in 136/240 (56.67%) CRC cases. NDRG1 expression was inversely correlated with pT (*p* = 0.013), pN (*p* = 0.012), pM (*p* = 0.027), pathological differentiation (*p* = 0.042) and clinical TNM stage (*p* = 0.011) (Table 5). Our data revealed that NDRG1 was highly expressed in the less aggressive tumors and had an inverse correlation with the well-established prognostic evaluation factors. Hence, NDGR1 could be used as a potential favorable prognostic biomarker in CRC.

**Table 5:** Correlation of NDRG1 expression with demographic and anatomopathological data.

		Patients Enrolled (N = 240)		
Parameters		NDRG1 (+) (n = 136)	NDRG1 (−) (n = 104)	*p*
**Age (Years)**	**20–39**	8	0	0.121
	**40–59**	22	28	
	**60–79**	90	58	
	**≥80**	16	18	
**Gender**	**Male**	92	60	0.266
	**Female**	44	44	
**BMI (kg/m^2^)**	**<18**	6	2	0.566
	**18–24**	62	56	
	**≥24**	68	46	
**ASA score**	**I**	10	8	0.796
	**II**	68	58	
	**III**	58	38	
**Tumor Location**	**Right-hemi Colon**	38	30	0.496
	**Transverse Conlon**	2	2	
	**Left-hemi Colon**	14	2	
	**Sigmoid**	28	26	
	**Rectum**	54	44	
**Tumor Size (cm)**	**<5**			0.137
	**≥5**	84	50	
**pT**	**pT1**	10	0	0.013
	**pT2**	18	2	
	**pT3**	64	50	
	**pT4**	44	52	
**pN**	**pN0**	102	52	0.012
	**pN1**	26	36	
	**pN2**	4	8	
**pM**	**pM0**	126	90	0.027
	**pM1**	6	18	
**Differentiation**	**Well**	20	6	0.042
	**Moderate**	82	58	
	**Poor**	12	22	
	**Mucinous**	7	14	
**TNM Stage**	**I**	28	2	0.011
	**II**	70	54	
	**III**	26	34	
	**IV**	14	10	

**Figure 3:**
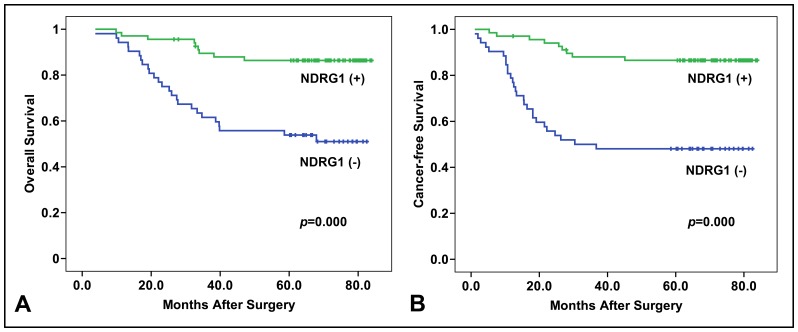
Overall survival and cancer-free survival analysis between NDRG1 positive and negative population. (A) Overall survival analysis between NDRG1 positive and negative group was performed by Kaplan-Meier method. The NDRG1 positive population had significant (*p* = 0.000) better overall survival than NDRG1 negative population. (B) Cancer-free survival analysis between NDRG1 positive and negative group was performed by Kaplan-Meier method. The NDRG1 positive population had significant (*p* = 0.000) better cancer-free survival than NDRG1 negative population.

We next investigated the correlation of NDRG1 expression with patient survival. As shown in Figure 3A, the 5-year overall survival rate in NDRG1 positive group was 82.35%, whereas in NDRG1 negative group, the 5-year overall survival rate was 51.92%. In addition, the estimated overall survival time was 76.4±2.4 months in NDRG1 positive group and 55.3±4.1 months in NDRG1 negative group. The NDRG1 positive group showed a significant better overall survival rate compared to the NDRG1 negative group (*p = *0.000) (Figure 3A).

The 5-year cancer-free survival rate in NDRG1 positive group was 80.89%. However, this was only 44.23% in NDRG1 negative group. In addition, the estimated overall survival time was 75.7±2.6 months in NDRG1 positive group and 47.2±4.8 months in NDRG1 negative group. The NDRG1 positive group showed a significant better cancer-free survival rate compared to the NDRG1 negative group (*p = *0.000) (Figure 3B). Thus, NDRG1 could be an ideal biomarker for survival and disease-free recovery in CRC patient.

### NDRG1 Expression can be Regarded as a Risk Factor for CRC Patient

As demonstrated in Table 6, univariate analysis indicated that pathological differentiation (*p* = 0.008), pT *(p* = 0.030), pN (*p* = 0.001), pM (*p* = 0.000), clinical stage (*p* = 0.001) and NDRG1 expression (*p* = 0.000) were found to be independent risk factors for overall survival. Tumor size (*p* = 0.044), pT (*p* = 0.006), pN (*p* = 0.014), clinical stage (*p* = 0.000) and NDRG1 (*p* = 0.000) were considered to be independent risk factors for recurrence.

**Table 6:** Univariate risk evaluations for recurrence and survival in colorectal cancer patients.

	Over-all Death Risk (n = 240)		Recurrence Risk (n = 216)	
Factors	F	*p*	F	*p*
**Age**	0.489	0.486	0.929	0.337
**Gender**	0.051	0.822	1.393	0.240
**BMI**	0.463	0.498	0.979	0.325
**ASA Score**	0.454	0.817	0.573	0.451
**Tumor Location**	2.167	0.077	1.606	0.178
**Tumor Size**	1.824	0.179	4.041	0.044
**Pathological Differentiation**	7.363	0.008	7.762	0.006
**pT**	4.853	0.030	6.223	0.014
**pN**	12.203	0.001	17.030	0.000
**pM**	45.582	0.000	/	/
**TNM Stage**	12.203	0.001	17.030	0.000
**NDRG1 Expression**	13.814	0.000	25.478	0.000

Similarly, multivariate analysis (Table 7) further indicated that pT (*p* = 0.002), pN (*p* = 0.007), pM (*p* = 0.004), clinical stage (*p* = 0.032) and NDRG1 (*p* = 0.001) were independent risk factors for overall survival. Nevertheless, only clinical stage (*p* = 0.021) and NDRG1 (*p* = 0.003) were the significant independent risk factors for recurrence. Taken together, NDRG1 is a promising independent biomarker for assessing disease relapse and survival in CRC patients.

**Table 7:** Multivariate risk evaluations for overall survival and recurrence in colorectal cancer patients.

	Overall Survival Risk (n = 240)			Recurrence Risk (n = 216)		
Factors	*p*	HR	95% CI	*p*	HR	95% CI
**Pathological Differentiation**	0.107	0.728	0.495–1.071	0.502	0.783	0.383–1.600
**pT**	0.002	3.464	1.600–7.501	0.910	1.039	0.536–2.013
**pN**	0.007	3.063	1.355–6.921	0.049	3.788	0.548–7.131
**pM**	0.004	3.106	1.438–6.711	/	/	/
**Tumor Size**	/	/	/	0.706	1.148	0.561–2.394
**TNM Stage**	0.032	0.482	0.247–0.940	0.021	2.619	1.234–5.863
**NDRG1 Expression**	0.001	3.732	1.718–8.108	0.003	2.821	1.418–5.612

## Discussion

Despite advances in surgical and nonsurgical therapies for CRC, metastasis and recurrence still remain the major challenges for surgeons and oncologists [4]. Recurrence and metastasis represent the main cause of death in CRC patients after radical surgeries [1], [29]. The clinical available biomarkers, including PCNA, CEA, CA19-9, p53, K-ras, MSI and VEGF, have been used for early detection and follow-ups for postoperative recurrence and metastasis [25]. Although these biomarkers may provide useful information for predicting patient outcome, there is a lack of good linear relationship with CRC metastasis and recurrence rate [25]. Therefore, developing novel prognostic biomarkers with higher specificity and sensitivity will benefit for CRC patients in therapeutic intervention.

Most of human solid tumors are characterized as high metabolic rate, active cell migration and invasion capacity and aberration of signaling transductions which play key roles in metastasis [30]. Given these traits in tumor cells, these cells are grown in an environment with a shortage of oxygen and nutrients until tumor angiogenesis or implantation to new environment [31], [32]. Thus, genes induced by hypoxia, such as NDRG1 [33], may affect cancer cell survival and metastasis. We and others demonstrated that NDRG1 is a novel tumor metastatic suppressor in different tumor cells including CRC [9], [10], [12]. In this study, the expression of NDRG1 in CRC patient specimens was investigated. We showed that NDRG1 expression was significantly reduced in CRC tissues compared to the paired non-tumor tissues, further supporting a tumor suppressive function of NDRG1 in CRC as previously reported [11], [22]. Similar results were also observed in pancreas, prostate, breast and esophageal carcinoma tissue where the NDRG1 mRNA and protein levels were both decreased compared to normal counterparts [21], [34]–[38]. In contrast, NDRG1 was found to be highly expressed in liver and cervical cancer tissues and its expression was associated with vascular invasion, metastasis, and poor prognosis [13], [16], [17]. It is plausible that NDRG1’s role in cancer progression is regulated by its potential mutations in different cancerous disease, leading to a “loss-of-function” or by the intrinsic genetic mechanism of the specific organs.

Several investigations have been reported referring to the detailed mechanisms of NDRG1 in inhibiting colorectal cancer migration, invasion and metastasis *in vitro* [9], [10], [12]. *NDRG1* mRNA as well as protein expression were reported to be over-expressed in normal human colon epithelial tissues [39]. In addition, Li and Chen reported that NDRG1 plays vital roles in tumor metastasis suppression and is frequently silenced in metastatic colon cancers [12]. They demonstrated a correlation between the increased histone H3S10p and silencing of the NDRG1 gene in colon cancer cell line SW620, suggesting a potential mechanism of NDRG1 repression during colon cancer metastasis. Recent studies demonstrated novel functions of NDRG1 in the inhibition of TGF-β-induced epithelial-mesenchymal transition (EMT) [9] and actin filament polymerization and stress fiber assembly through modulating the ROCK1/pMLC2 pathway in CRC cell lines [10]. These findings revealed potential mechanisms underlying the tumor suppressive function of NDRG1 in CRC observed in this study.

Previous studies indicated that the decreased NDRG1 expression was independent unfavorable prognostic factors for survival of patients with high risk stage II colorectal cancer [22]. In supporting of this, we included 240 CRC specimens with various TNM stages (I-IV) to investigate the clinical potential of NDRG1 alone as an independent prognostic factor. In these CRC cases who underwent laparoscopic procedures, NDRG1 expression inversely correlated with pT, pN, pM and TNM stage. Intriguingly, Kaplan-Meier analysis showed that CRC patients with NDRG1 negative tumors had significant worse prognosis in both overall survival and cancer-free survival. Hence, NDRG1 was an independent prognostic factor for both overall survival and recurrence and its positivity could be a favorable prognostic biomarker for clinical application in CRC patients. Given the importance of other known biomarkers including PCNA, K-ras and MSI in CRC, it is of clinical significance to examine the association of NDRG1 with these biomarkers in CRC patients.
